# Recombinant Human Endostatin Endostar Suppresses Angiogenesis and Lymphangiogenesis of Malignant Pleural Effusion in Mice

**DOI:** 10.1371/journal.pone.0053449

**Published:** 2012-12-28

**Authors:** Xingqun Ma, Yanwen Yao, Dongmei Yuan, Hongbing Liu, Shouju Wang, Changsheng Zhou, Yong Song

**Affiliations:** 1 Department of Respiratory Medicine, Jinling Hospital, Nanjing University School of Medicine, Nanjing, China; 2 Department of Medical Imaging, Jinling Hospital, Nanjing University School of Medicine, Nanjing, China; Cincinnati Children's Hospital Medical Center, United States of America

## Abstract

**Background:**

Malignant pleural effusion (MPE) is a common complication of lung cancer. One widely used treatment for MPE is Endostar, a recombined humanized endostatin based treatment. However, the mechanism of this treatment is still unclear. The aim of this study was to investigate the effects of Endostar in mice with MPE.

**Methods and Materials:**

Lewis lung carcinoma (LLC) cell line expressing enhanced green fluorescent protein (EGFP) was injected into pleural cavity to establish MPE mice model. Mice were randomly divided into four groups. High dose of Endostar (30 mg/kg), low dose of Endostar (8 mg/kg), normal saline, or Bevacizumab (5 mg/kg) was respectively injected into pleural cavity three times with 3-day interval in each group. Transverse computed tomography (CT) was performed to observe pleural fluid formation 14 days after LLC cells injection. Mice were anesthetized and sacrificed 3 days after final administration. The volume of pleural effusion n was measured using 1 ml syringe. Micro blood vessel density (MVD), Lymphatic micro vessel density (LMVD), the expression level of vascular endothelial growth factor A (VEGF-A) and VEGF-C were observed by immunohistochemistry (IHC) staining.

**Results:**

The volume of pleural effusion as well as the number of pleural tumor foci, MVD and the expression of VEGF-A were significantly reduced in high dose of Endostar treat group. More importantly, LMVD and the expression of VEGF-C were markedly lower in treat group than those in the other three control groups.

**Conclusion:**

Our work demonstrated that Endostar played an efficient anti-cancer role in MPE through its suppressive effect on angiogenesis and lymphangiogenesis, which provided a certain theoretical basis for the effectiveness of Endostar on the MPE treatment.

## Introduction

Lung cancer is the leading cause of cancer-associated mortality in the world. Most lung cancer patients are diagnosed at late stage and more than 20% of patients have malignant pleural effusion (MPE) when diagnosed [1]. Lung cancer patients with MPE are associated with poor survival and quality of life [2]. Although MPE is common in clinic, the causes and related mechanisms are still not clear. Existing research has revealed that lung adenocarcinoma is the most common histological type responsible for MPE and angiogenesis is considered to be associated with MPE formation [3], [4]. However, some studies showed that solely suppressing angiogenesis cannot reduce the formation of MPE [5]. Compared with blood vessel, lymphatic vessel has larger lumen and increased permeability, which leads cancer cells to spread through lymph system more easily [6]. Previous studies demonstrated that impaired lymphatic circulation is considered to be another primary mechanism for MPE formation [7]. Lymphatic vessels can be blocked directly by tumors on the parietal pleura and enlarged mediastinal nodes can lead lymphatic return, which then disrupt the lymphatic circulation and force the MPE formation. Suppressing lymphangiogenesis on MPE formation may provide another therapy strategy for lung cancer patients with MPE [8].

Endostatin, a proteolytic C-terminal fragment of the vascular and epithelial basement membrane collagen type XVIII, has been proven to be efficient in anti-angiogenesis and tumor inhibitor [9]. Previous studies demonstrated that endostatin overexpression inhibited lymphangiogenesis and lymph node metastasis in mice via down-regulating vascular endothelial growth factor (VEGF)-C gene expression [10], [11]. In MPE treatment, Talc was revealed to play an inhibitory role via endostatin induction [12]. However, whether endostatin has effect on MPE by anti-lymphangiogenesis and suppressing lymph node metastasis is not elucidated yet.

Since recombinant human endostatin are more efficient than original endostatin, it is now widely used in clinic [13]. Endostar, a recombinant human endostatin with an additional nine-amino acid sequence (MGGSHHHHH) added to the N-terminal of the protein, is a common angiogenesis antagonist for lung cancer patients [14].

The present study was designed to investigate the effect of Endostar on MPE mouse model. We also aim to explore whether the involved mechanism is associated with both angiogenesis and lymphangiogenesis.

## Materials and Methods

### Mice

Age (6–8 weeks) -, weight (19–27 g) - and sex (male)-matched nude mice were used for the MPE studies. All the mice were BALB/c background. Animal care and experimental procedures were approved by Model Animal Research Centre of Jingling Hospital and conducted according to Institutional Animal Care and User guidelines.

### Cell Line, Culture, and Transfection

The LLC-EGFP cell line was purchased from American Type Culture Collection (ATCC). The Lewis lung cancer (LLC) cell line (ATCC) was cultured in RPMI 1640 medium (Hyclone) containing 10% fetal bovine serum (Hyclone), penicillin (100 U/mL) and streptomycin (100 ug/mL) (Gibco). Cells were cultured at 37°C in 5% CO_2_.

### MPE Model and Treatment Process

Mice were anesthetized using ketamine and a 5mm-long vertical cut was made on the right side of manubrium sterni. Skin and subcutaneous fascia were retracted without damage of intercostal muscles. A total of 5×10^5^ EGFP-LLC cells suspended in 50 ul PBS was pipetted with micropipettor and injected into pleural cavity via intercostal space under the guidance of stereo microscope.The depth of needle penetration was about 3–5 mm to avoid piercing the visceral pleura or lung. After the injection, the wound was sutured. No mortality or morbidity was associated with the procedure [15]. According to the methods provided by Dong et al. [16] and Fang Fang et al. [17], three days after EGFP-LLC cells injection, mice were randomly divided into four groups, 10 mice each group, treated with normal saline (NS), Bevacizumab (Roche, China), low dose of Endostar (L-ES) (Simcere, China) and high dose of Endostar (H-ES), respectively. The treat group (H-ES group) was administered with 30 mg/kg of Endostar (according to the preliminary experiment) three times with the 3-day interval, another three control groups (NS, Bevacizumab, L-ES group) were administered with 50 ul of normal saline, 5 mg/kg of Bevacizumaband 8 mg/kg of Endostar, respectively [17]–[19]. The treatment processes in control groups were the same as H-ES group.

### Computed Tomography Scanning

As described previously [17], 14 days after LLC-EGFP cells injection, computed tomography (CT) images were acquired (Siemens Somatom Sensation 16, 120 kVp, 93 µA) for the observation of pleural effusion. Mice were anesthetized as described previously during the imaging session. Scanned images were real-time transferred to multi-functional image post-processing workstation (Syngo MMWP CT workplace VA30A).

### Pleural Effusion Measurement and Tumor Foci Counting

Three days after the final administration as described above, mice were anesthetized and sacrificed. Pleural effusion was gently aspirated using 1 ml syringe, and the volume was measured. The pleural cavity of mice was opened up and the expression of EGFP in tumors was observed using fluorescence imaging system, which helped to detect the distribution of tumor lesions [20]. Pleural tumors scattered on visceral and parietal pleural surfaces as well as mediastinal and pulmonary hilar lymph nodes. Therefore, the total number of tumor lesions was independently counted by two investigators (MA and YAO). The numbers from two investigators were compared and disagreements were resolved by consensus.

### Hematoxylin-Eosin Staining and Cytology Assay

Tumors from parietal pleura were acquired and fixed in 10% formalin for 24 hours, following 70% ethanol for 3 days and embedded in paraffin, finally stained with Hematoxylin-Eosin. Acquired pleural effusion from NS treat group was tested for cytology. Pleural effusion was centrifuged and the cells in effusion were applied on the slides, air dried, fixed in methanol for 10s and stained with modified Wright’s Giemsa stain [21].

### Immunohistochemistry Staining

Angiogenesis and lymphangiogenesis were tested by immunohistochemistry (IHC) staining. Tumors from parietal pleura were fixed in 10% formalin for 24 hours and embedded in paraffin after routine dehydration. Four consecutive slides from each tissue were stained for micro blood vessel density (MVD), lymphatic micro vessel density (LMVD), VEGF-A, VEGF-C analysis. Briefly, tissues were incubated with a polyclonal goat anti-mouse CD31 antibody (1∶200, Abcam), mouse monoclonal D2-40 antibody (1∶200, Abcam), polyclonal goat anti-mouse VEGF-A antibody (1∶200, Abcam) and polyclonal goat anti-mouse VEGF-C antibody (1∶200, Abcam), respectively at 4°C overnight. Positive reaction was defined as brown using 3, 3-diamino-benzidine (DAB) (Vector Laboratories) and nuclear was restained with hematoxylin [22], [23]. Staining for CD31 was used to evaluate MVD, assessed by counting all stained vessels at ×200 magnification. The mean number of vessels was defined as MVD [22]. Staining for D2–40 was used to evaluate LMVD and the method was the same as MVD. Positive staining of VEGF-A and VEGF-C expression was defined according to previous studies [24]. Staining intensity was given with four grades: none (0), weak (1), moderate (2) and strong (3). The percentage of positive carcinoma cells was also given with four grades: 0(0%), 1(1%∼10%), 2(11%∼49%), 3(50%∼100%), respectively. Staining result was semi-quantitatively assessed by the score combined with staining intensity and the percentage of positive cells [24]. Tumors were categorized into low-expression group (score = 0∼3) and high-expression group (score = 4∼6). The scores from two independent investigators were compared and disagreements were resolved by consensus.

### Statistical Analysis

All statistical analyses were performed using the computing environment R. All values represent mean ± SE, Kruskal-Wallis analysis (Dunn’s test) was used to test the differences in the means between two or multiple groups. All *P* values are two-tailed; *P* values <0.05 were considered significant.

## Results

### Treatment with Endostar Inhibited MPE Formation and Tumor Growth

To determine the therapeutic effect of Endostar on MPE formation, MPE mouse model was established by using EGFP-LLC cell line and administered with H-ES, L-ES, Bevacizumab and NS.

**Figure 1:**
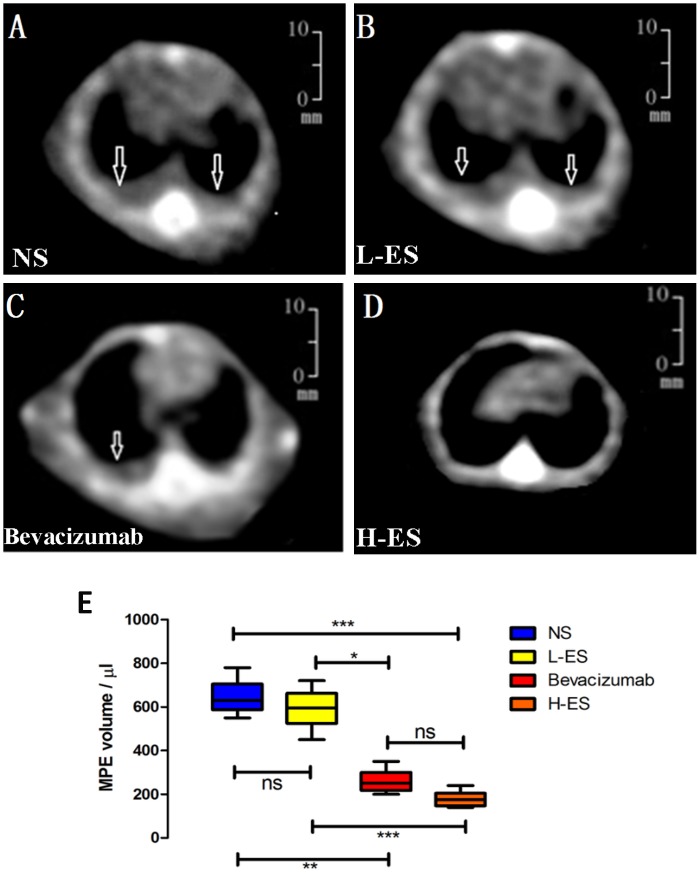
CT scanning of MPE formation in four groups. CT images of four groups showed that bilateral pleural effusion was visible in the mice treated with NS (A) or L-ES (B),unilateral pleural effusion was observed in Bevacizumab group (C), and effusion was not obvious in H-ES group (D). The mean volume of pleural effusion was significantly decreased in the H-ES group compared with that in the NS group or L-ES group, but there is no significant difference between H-ES group and Bevacizumab group (E). MPE: malignant pleural effusion. Columns: mean value of each group, bars: ±SD. ***P<0.001, **P<0.01, *P<0.05. ns: no significant difference.

Fourteen days after intra-pleural tumor cells injection, bilateral pleural effusion on CT scanning was more visible in mice administered with NS or L-ES, unilateral pleural effusion was observed in Bevacizumab group, and no obvious pleural effusion was observed in H-ES group, as shown in Figure 1A–D. Pleural effusion from four groups all appeared to be hemorrhagic and non-coagulated. The mean volume of pleural effusion in NS, L-ES, Bevacizumab and H-ES group was 643±71.81 ul, 594±92.52 ul, 260±46.93 ul and 178±33.60 ul, respectively. The volume of pleural effusion was reduced in the H-ES group, significantly different from that in NS group (P<0.001) and L-ES group (P<0.01), but the difference between H-ES group and Bevacizumab group was no obvious (Figure 1E).

**Figure 2:**
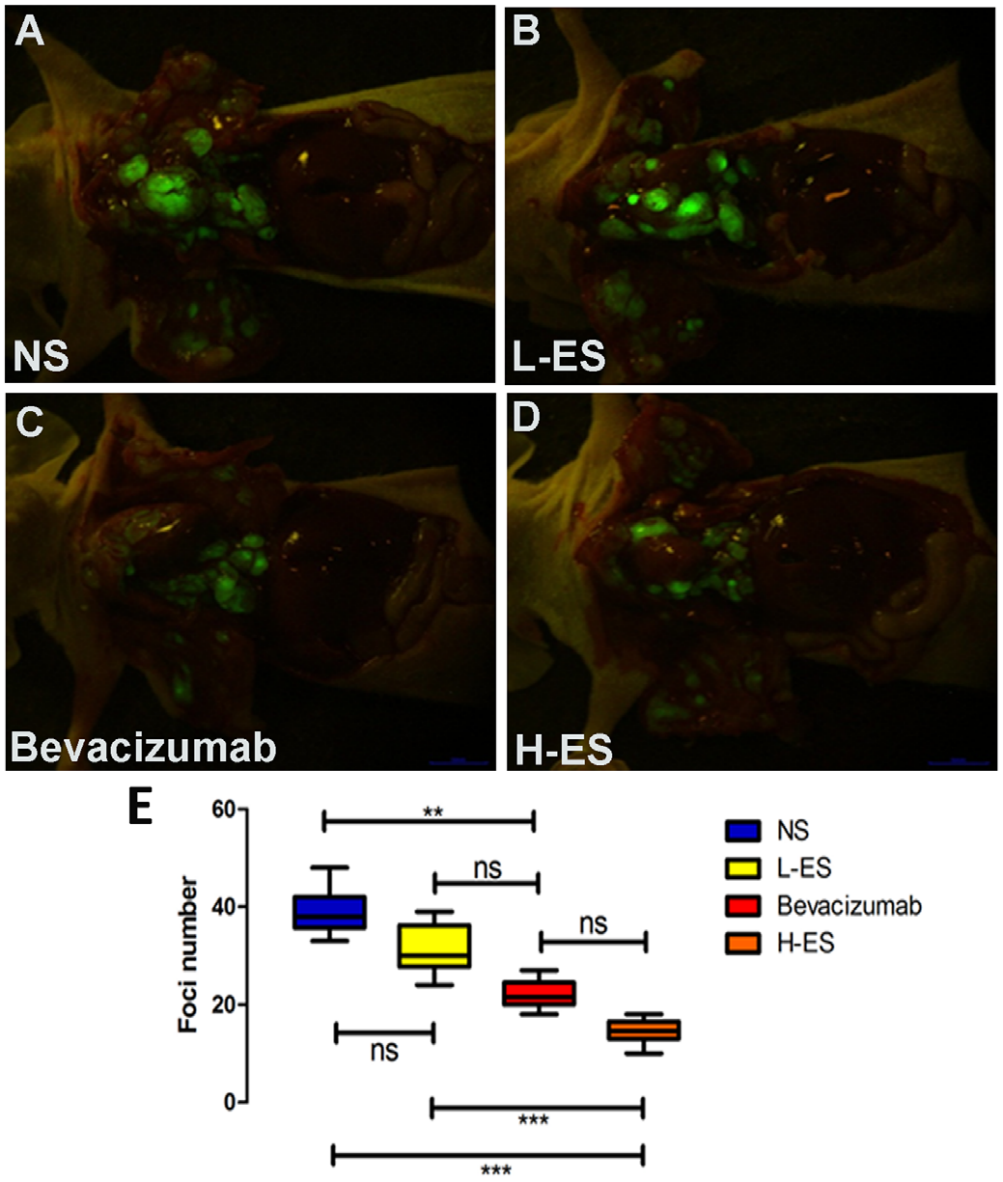
Tumor foci from four groups under fluorescence imaging system. Mice were sacrificed and scanned by fluorescent imaging system. Fluorescent tumor foci were observed on the parietal and visceral pleura as well as hilar and mediastinal lymph node. The number of fluorescent pleural tumor loci was significantly decreased in H-ES group (D) compared with that in NS group (A) and L-ES group (B). The number of fluorescent pleural tumor loci in Bevacizumab group (C) was similar with that in H-ES group (D). (E): The difference of the number of Tumor foci on mice from four groups. Columns: mean value of each group, bars: ±SD. ***P<0.001, **P<0.01, *P<0.05. ns: no significant difference.

Compared to general picture, fluorescence imaging is more visible and accurate to detect micro-metastatic tumors. EGFP expressed tumors scattered on visceral and parietal pleura as well as hilar and pulmonary mediastinal lymph node. As shown in Figure 2A–D, the number of tumors in NS, L-ES, Bevacizumab and H-ES group was 38.8±4.39, 31.3±4.92, 22.1±2.88 and 14.4±2.76, respectively. The number of tumor foci in H-ES group was notably reduced compared with that in NS group (P<0.001), L-ES (P<0.001) group and still not significantly different with Bevacizumab group (Figure 2E).

**Figure 3:**
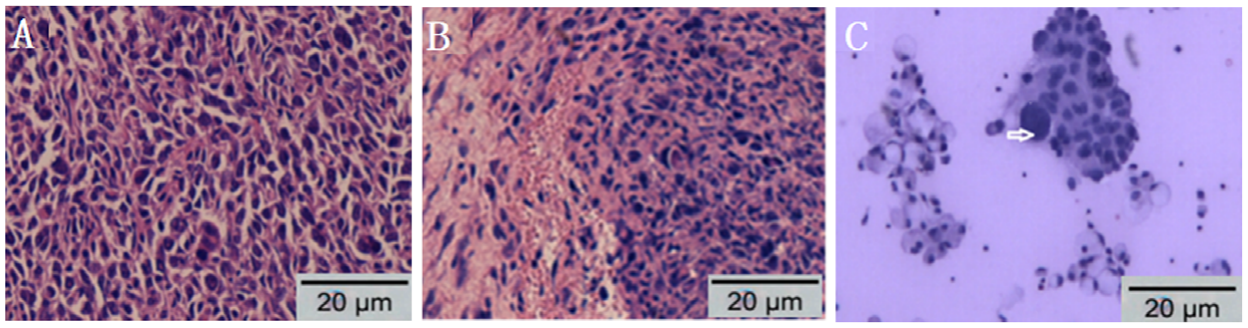
Histology of pleural tumors and cytology of MPE from the mice in NS group. (A) Hematoxylin-eosin staining of parietal pleura from MPE model (Section ×200) indicated that pleural tumors consisted of adenocarcinomatous cells. (B) Hematoxylin-eosin staining of tumor on the pleural surface from MPE model (Section ×200). (C) Wright’s-Giemsa stain of cells from pleural effusion of MPE model showed LLC cells with large nuclei and visible nucleoli (arrow). MPE: malignant pleural effusion.

To avoid the influence of drugs, pleural tumors on parietal pleural were taken from the mice in NS group and observed by histology staining. The result of staining confirmed that tumors were consisted of adenocarcinoma cells (Figure 3A, B) and cytology of pleural effusion from NS group revealed that LLC cells with large nuclei and visible nucleoli, (Figure 3C).

### Treatment with Endostar Inhibited Tumor Angiogenesis and VEGF-A Expression

**Figure 4:**
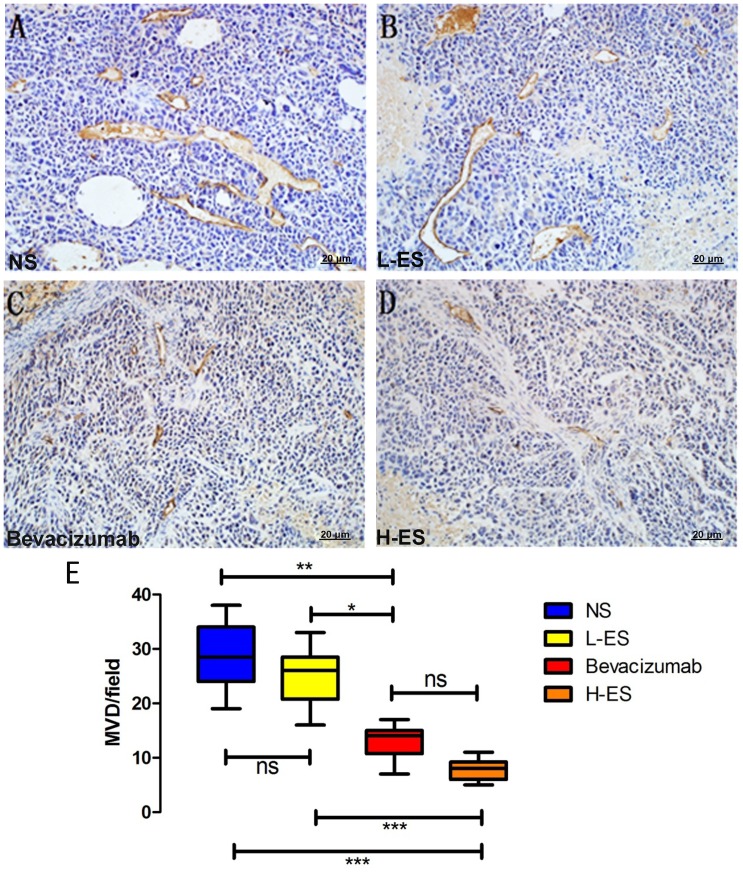
Immunohistochemistry staining of CD31 for MVD in the pleural tumors. Positive immunohistochemistry staining of CD31 was shown as brown part in each figure. Micro-vessel density (MVD) was counted at Section×200. Well-formed capillaries were observed in the tumors from NS group (A) and L-ES group (B). Isolated micro-vessels were shown in Bevacizumab group (C) and H-ES group (D). MVD was significantly decreased in H-ES group compared with that in NS group or L-ES group, and there is no significant difference between Bevacizumab group and H-ES group (E).Columns: mean value of each group, bars: ±SD. ***P<0.001, ** P<0.01, *P<0.05. ns: no significant difference.

To verify Endostar affect MPE through inhibiting angiogenesis, MVD of tumors from pleura was measured by IHC staining for CD31. The mean value of MVD in NS, L-ES, Bevacizumab and H-ES group was 28.8±6.27, 24.9±5.47, 13±2.90 and 7.8±1.87, respectively, which is shown in Figure 4A–D. In H-ES group, MVD was dramatically decreased, compared with NS group (P<0.001) or L-ES group (P<0.001). Similar results were observed in Bevacizumab group compared with NS group (P<0.01) and L-ES group (P<0.05). The difference between H-ES group and Bevacizumab group was not significant.

**Figure 5:**
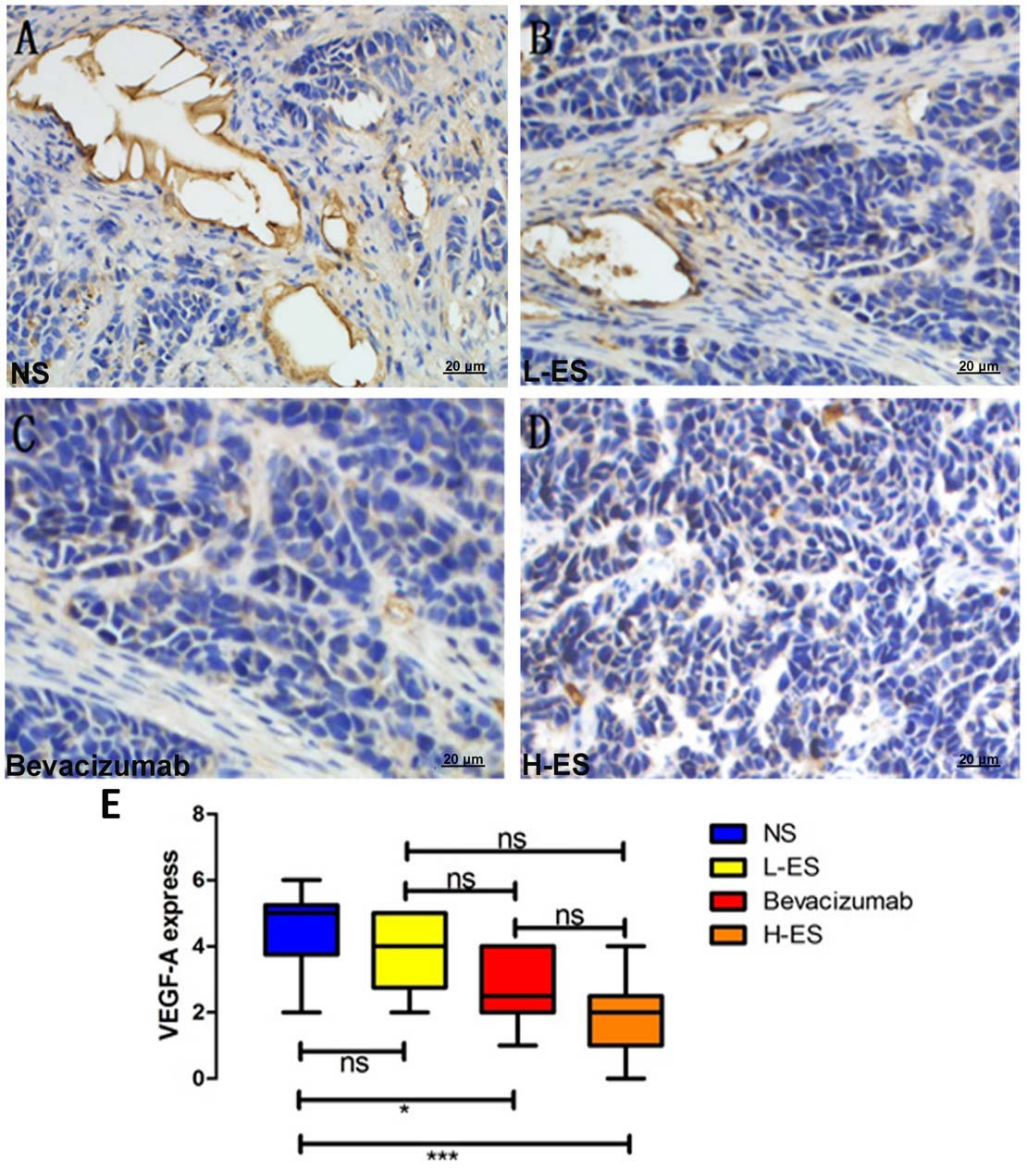
Immunohistochemistry staining of VEGF-A expression in the pleural tumors. Positive immunohistochemistry staining of VEGF-A was shown as brown part in each figure. Expression of VEGF-A was accessed by the percentage of positive carcinoma cells and the staining intensity. The positive staining of VEGF-A in NS group (A) and L-ES group (B) indicated high expression of VEGF-A in these groups. Low expression of VEGF-A was shown in Bevacizumab group (C) and H-ES group (D). The expression of VEGF-A was significantly decreased in H-ES group compared with that in NS group or L-ES group, and there is no significant difference between Bevacizumab group and H-ES group (E). Columns: mean value of each group, bars: ±SD. ***P<0.001, **P<0.01, *P<0.05. ns: no significant difference.

Previous studies have demonstrated that VEGF-A is one of the most important factors regulating angiogenesis. Therefore, VEGF-A expression in tumors was detected by IHC staining. According to the total IHC sores described above, High VEGF-A expression was observed in NS group (Figure 5A) and L-ES group (Figure 5B) and low VEGF-A expression was shown in Bevacizumab group (Figure 5C) and H-ES group (Figure 5D). Compared with NS group, VEGF expression in H-ES group and Bevacizumab group was decreased, but there was no significant difference between H-ES group and Bevacizumab group (Figure 5E).

### Treatment with Endostar Inhibited Tumor Lymphangiogenesis and VEGF-C Expression

To further investigate the mechanism of H-ES in inhibition of MPE formation, LMVD and VEGF-C expression were also assessed.

**Figure 6:**
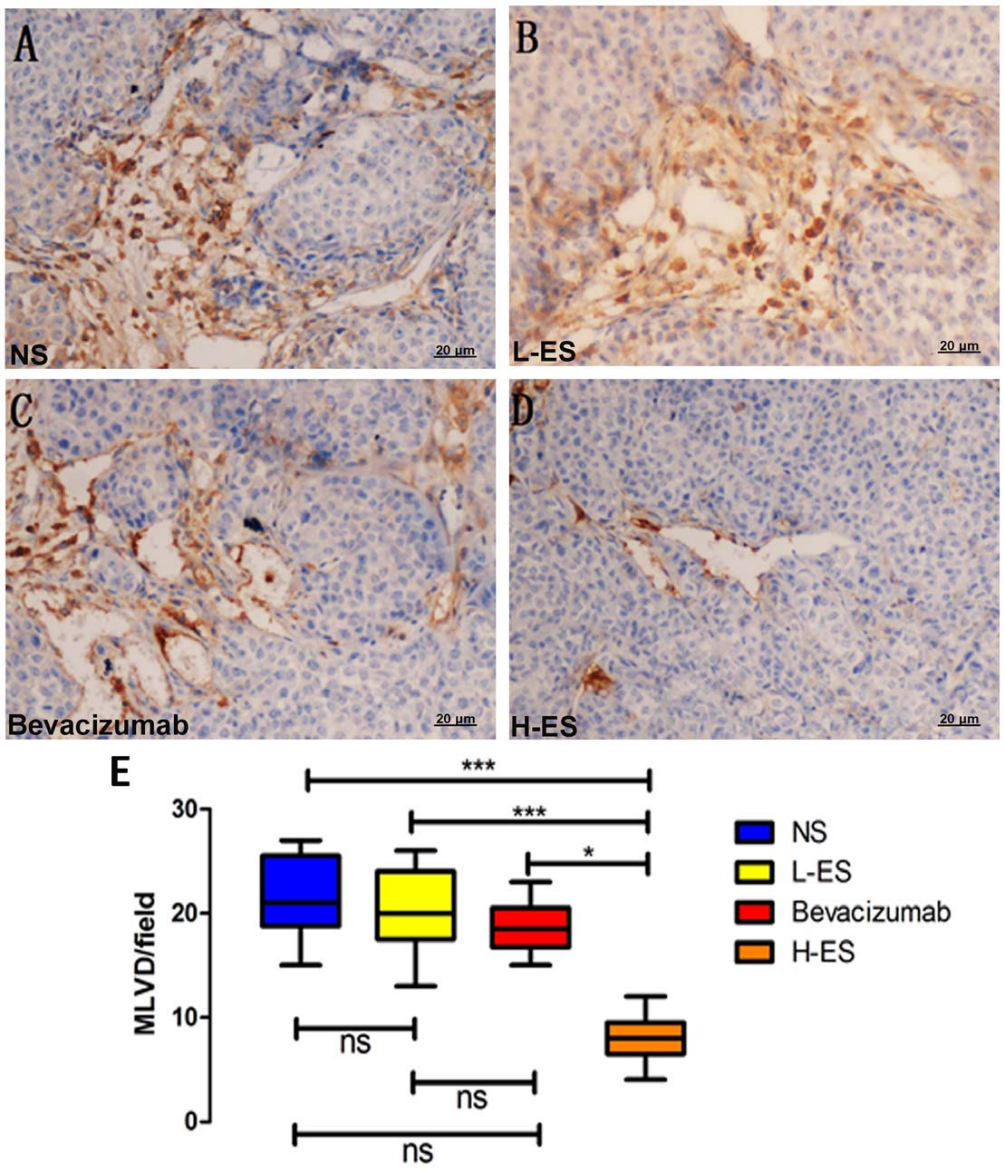
Immunohistochemistry staining of D2-40 for LMVD in the pleural tumors. Positive immunohistochemistry staining of D2-40 was shown as brown part in each figure. Positive endothelial cells stained by anti-D2-40 antibody were recognized as lymphatic vessels. Lymphatic micro vessel density (LMVD) was counted at Section×200. LMVD in H-ES group (D) was significantly decreased compared with NS group (A) or L-ES group (B) or Bevacizumab group(C). (E): The difference of LMVD on four groups. Columns: mean value of each group, bars: ±SD. ***P<0.001, **P<0.01, *P<0.05. ns: no significant difference.

The mean value of LMVD in NS, L-ES, Bevacizumab and H-ES group were 21.6±3.95, 20±3.92, 15.7±2.36 and 8.1±2.42, respectively (Fig. 6A–D). MVD was significantly reduced in H-ES treat group compared with NS group (P<0.001), L-ES group (P<0.001) and Bevacizumab group (P<0.05). No statistically difference was observed between the three control groups, shown in Figure 6E.

**Figure 7:**
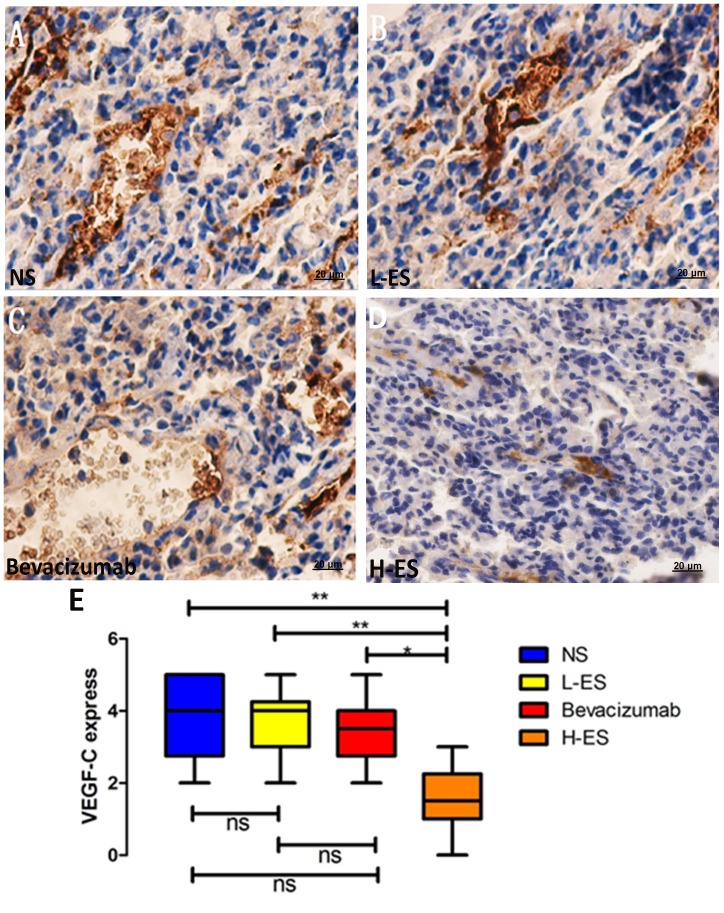
Immunohistochemistry staining of VEGF-C expression in the pleural tumors. Positive immunohistochemistry staining of VEGF-C was shown as brown part in each figure. Expression of VEGF-C was accessed by the percentage of positive carcinoma cells and the staining intensity. The positive staining of VEGF-C in NS group (A) and L-ES group (B) indicated high expression of VEGF-C in these groups. Low expression of VEGF-C was shown in Bevacizumab group (C) and H-ES group (D). The expression of VEGF-C was significantly decreased in H-ES group compared with that in NS group or L-ES group or Bevacizumab group.Columns: mean value of each group, bars: ±SD. ***P<0.001, **P<0.01, *P<0.05. ns: no significant difference.

VEGF-C expression in NS group, L-ES group, Bevacizumab group and H-ES group was 70% (7/10), 60% (6/10), 50% (5/10) and 30% (3/10), respectively. IHC staining for VEGF-C in each group is shown in Figure 7A to D. Significantly decrease was demonstrated in H-ES treat group compared with the other three control groups treated with NS (P<0.01) or L-ES (P<0.05) or Bevacizumab (P<0.05), and there was no significant difference between three control groups (Figure 7E).

## Discussion

MPE is a common complication in advanced malignancies, particularly in lung and breast cancer [7]. The survival time of patients with MPE is often short and lack of effective treatment [3]. A curative treatment for MPE needs a better understanding of the biologic processes that drive MPE, which can provide an effective target to prevent or inhibit MPE.

Stathopoulos created a MPE mouse model by inter-pleural injection of LLC cells through intercostal space [21], [25]. But skin, fascia and muscle on the pleura were retracted, induce the intercostal artery easily damaged and lead to infection. In current study, according to the preliminary experiment, we established our MPE mouse model under the guidance of the stereo microscope without retraction the intercostal muscle so that the LLC cells could be visually given into the thoracic cavity. And we were first to give LLC-EGFP cells into the pleural cavity to establish the MPE model. Under the fluorescence imaging, we can visually and directly observe tumor colonization, growth and migration in the pleural cavity avoiding the false-positive results. Therefore, the MPE model established in our study pave the way for exploration of the mechanism of MPE in lung cancer.

Research and clinical trials show evidence that endostatin is an efficient tumor inhibitor for lung cancer and the mechanism of the therapeutic effect is predominantly through angiogenesis inhibition [9]. As an improved recombinant endostatin, Endostar is now widely used in clinic for lung cancer patients [14]. However, no systematic study of Endostar for MPE has been reported. In our study, we established mouse model of MPE and treated MPE by Endostar injected into pleural cavity compared with Bevacizumab and Placebo. Both pleural effusion and tumor loci on pleura were decreased in high dose of Endostar group compared with placebo or low dose of Endostar group. The Results suggested that Endostar had therapeutic effect on MPE of lung cancer and the effect was dose-dependent. Bevacizumab is a recombinant humanized monoclonal anti-VEGF antibody that specifically binds VEGF-A to disrupt its ability to activate its receptors. The main antiangiogenic mechanism of bevacizumab is thought to result from its blockade of VEGF-mediated activation of VEGFR2 in endothelial cells [26]–[28]. Bevacizumab was also demonstrated anti-MPE effect in other studies [29]. Notably, pleural effusion in H-ES treatment group was less than Bevacizumab group, which meant that the therapeutic effect of high dose of Endostar was comparable to Bevacizumab, although the difference was not significant.

The VEGF family includes five members: VEGF (also known as VEGF-A), VEGF-B, VEGF-C, VEGF-D and placenta growth factor (PIGF) [30]. Alternative splicing of the VEGF gene yields five isoforms, ranging from 121 to 206 amino acids [27]. The VEGF ligands mostly bind with three endothelial transmembrane tyrosine kinase receptors, VEGFR-1, VEGFR-2 and VEGFR-3. VEGF signaling through VEGFR-2 is the major pathway which activates angiogenesis [31]. VEGF-C induces lymphangiogenesis via VEGFR-3 and has also been shown to induce lymphangiogenesis in tumors and subsequently stimulate metastasis. Mouse models of lymphoedema have established that VEGF-C is a promising agent for pro-lymphangiogenic therapy [30], [32].

Since previous studies have demonstrated the mechanism of Endostar on angiogenesis was primarily through down-regulated VEGF-A expression [33], markers of angiogenesis were also observed in our studies. MVD and expression of VEGF-A in the high dose of Endostar **t**reat group or Bevacizumab treat group were notably decreased than that in placebo group. Results from our studies verified that Endostar suppressed MPE formation and tumor growth through inhibiting angiogenesis and the effect was dose-dependent and comparable to Bevacizumab.

In our study, we observed that the pleural effusion in H-ES group was less than Bevacizumab group, which may provide another mechanism of Endostar on MPE. Previous research revealed that endostatin could suppress tumor metastasis via inhibiting lymphangiogenesis and lymph node metastasis. Fukumoto et al. demonstrated endostatin gene overexpression inhibited tumor growth of oral squamous cell carcinoma and also inhibited lymph node metastasis in orthotopic implantation [11]. Brideau et al. also observed that over-expressing endostatin gene in mice skin tumor model could down-regulate VEGFR-3 and VEGF-C expression and inhibit lymphangiogenesis and lymph node metastasis [10]. To further investigate the mechanism, we detected the markers of lymphangiogenesis in tumors from four groups. Notably, both LMVD and VEGF-C expression were decreased in H-ES group. Compared with Bevacizumab group or placebo group, the difference was significant. The results demonstrated that besides inhibiting angiogenesis, high dose of Endostar also suppressed MPE through down-regulating VEGF-C to inhibit lymphangiogenesis.

There are still some shortages in our study. First, it was difficult to identify all metastatic lymph nodes from tumor lesions, so the tumor loci we counted may be the combination of primary tumors and metastasis lymph nodes. However, either from the primary tumor growth or lymph nodes metastasis, the decreased tumor loci still demonstrated the inhibition effect of Endostar.

Second, the time of CT scanning to confirm the pleural effusion was a little late, so we cannot tell the failed model establish from the effective treatment. But if we gave the treatment after CT confirmed pleural effusion, the time would be too late and mice may be died of MPE. So we applied 10 mice in each group to reduce the effect to small. Although there were these shortages, our study still provided a positive and reliable result on the effect of Endostar on MPE and the mechanism on anti-angiogenesis and anti-lymphangiogenesis.

### Conclusion

Taken together, in the present study, we first established a MPE mouse model by intra-pleural injection of EGFP-LLC cells and demonstrated the therapeutic effect of high dose of Endostar on MPE, which was comparable to Bevacizumab. And we further revealed the mechanism of Endostar, which inhibited angiogenesis and lymphangiogenesis through down-regulating the expression of VEGF-A and VEGF-C, respectively. It is the first study reported the anti-lymphangiogenesis effect of Endostar on MPE. Our results provided a certain theoretical basis for the effectiveness of Endostar on the MPE treatment. To confirm these findings, large, prospective, randomized clinical studies are required.
